# Atypical Auditory Perception Caused by Environmental Stimuli in Autism Spectrum Disorder: A Systematic Approach to the Evaluation of Self-Reports

**DOI:** 10.3389/fpsyt.2022.888627

**Published:** 2022-06-09

**Authors:** Jyh-Jong Hsieh, Yukie Nagai, Shin-ichiro Kumagaya, Satsuki Ayaya, Minoru Asada

**Affiliations:** ^1^Institute for Open and Transdisciplinary Research Initiatives, Osaka University, Osaka, Japan; ^2^International Research Center for Neurointelligence, The University of Tokyo, Tokyo, Japan; ^3^Institute for AI and Beyond, The University of Tokyo, Tokyo, Japan; ^4^Research Center for Advanced Science and Technology, The University of Tokyo, Tokyo, Japan; ^5^International Professional University of Technology in Osaka, Osaka, Japan; ^6^Chubu University Academy of Emerging Sciences, Kasugai, Japan; ^7^Center for Information and Neural Networks, National Institute of Information and Communications Technology, Osaka, Japan

**Keywords:** autism spectrum disorder, auditory perception, hyperesthesia, hypoesthesia, self-report

## Abstract

Recent studies have revealed that atypical sensory perception is common in individuals with autism spectrum disorder (ASD) and is considered a potential cause of social difficulties. Self-reports by individuals with ASD have provided great insights into atypical perception from the first-person point of view and indicated its dependence on the environment. This study aimed to investigate the patterns and environmental causes of atypical auditory perception in individuals with ASD. Qualitative data from subject reports are inappropriate for statistical analysis, and reporting subjective sensory experiences is not easy for every individual. To cope with such challenges, we employed audio signal processing methods to simulate the potential patterns of atypical auditory perception. The participants in our experiment were able to select and adjust the strength of the processing methods to manipulate the sounds in the videos to match their experiences. Thus, the strength of atypical perception was recorded quantitatively and then analyzed to assess its correlation with the audio-visual stimuli contained in the videos the participants observed. In total, 22 participants with ASD and 22 typically developed (TD) participants were recruited for the experiment. The results revealed several common patterns of atypical auditory perception: Louder sounds perceived in a quiet environment, noise perception induced by intense and unsteady audio-visual stimuli, and echo perception correlated with movement and variation in sound level. The ASD group reported atypical perceptions more frequently than the control group. However, similar environmental causes were shared by the ASD and TD groups. The results help us infer the potential neural and physiological mechanisms of sensory processing in ASD.

## Introduction

Autism spectrum disorder (ASD) is traditionally characterized by deficits in social communication and restricted and repetitive behaviors or interests (1). Additionally, recent studies have revealed that sensory issues involving haptic (2, 3), auditory (4), and visual perception (5) are common among individuals with ASD (6–8). Hyperreactivity or hyporeactivity to sensory stimuli is the most well-known symptom of ASD and has been included as a diagnostic criterion in the latest edition of the Diagnostic and Statistical Manual of Mental Disorders (1).

Individuals with ASD exhibit reduced sound tolerance (9). Some have reported that they are distressed by sounds or colors that others can ignore (10, 11). Further, hyposensitivity to bodily stimuli has been reported. Individuals with ASD may not notice hunger or thirst even when they have had a visibly negative impact on their physical condition (12, 13). Another study showed that individuals with ASD scored lower on pain and discomfort levels from electrical stimulation than the control group, although the pain detection threshold was not significantly different between the groups (14). In some cases, hypersensitivity and hyposensitivity can be regarded as two sides of the same coin. For example, several sensory inputs simultaneously may become an information overload for individuals with ASD, making them overwhelmed and unable to respond (10, 11). They are more sensitive to sensory inputs but exhibit fewer responses. Moreover, autistic traits are correlated with atypical sensory properties (15). Some researchers have even suggested that sensory issues may be a critical cause of social deficits in individuals with ASD (12, 16–18).

The characteristics of sensory processing for individuals with ASD have been quantitatively investigated in behavioral research. For instance, Bertone et al. (19) found that children with high-functioning autism exhibited a higher ability to identify luminance-defined orientation contrast and a lower ability to identify texture-defined contrast than those with typical development.

A higher motion coherence threshold has been reported in individuals with ASD (20, 21). The participants watched moving dots on a computer screen. A variable proportion of target dots moved coherently in either the left or right direction, while the remaining dots moved randomly. A larger proportion of coherently moving dots was required for the participants with ASD to recognize the direction of the coherent motion. Regarding studies of auditory perception, audiometric tests showed that individuals with ASD are superior to matched subjects at discriminating auditory stimuli with different frequencies (22, 23). Tolerance of intense sounds was found to be lower for individuals with ASD (9). Another study showed that loudness adaptation, which is a reduction in the loudness of a steady sound over time, to quiet steady-state sounds is reduced in individuals with ASD (24). Studies have evaluated the ability to process speech or human voices to investigate the relationship between communication difficulties and auditory perception. Individuals with high-functioning ASD perform less well than their typically developed peers in identifying speech in multi-talker babble noise, which consists of the sounds of four people reading non-sense text, but not from sounds of more than eight people reading non-sense text (25, 26). Lin et al. (27) showed that adults with ASD were faster at recognizing human voices than neurotypical adults, but there was no significant difference in recognizing string sounds (violin or cello). These behavioral studies have unveiled how individuals with ASD respond to sensory stimuli and provide objective comparisons to their typically developed peers.

Qualitative research, such as focus groups, interviews, and autobiographical self-reports, has provided evidence of sensory experiences from the viewpoint of individuals with ASD and helped us understand their needs (16, 28, 29). These studies revealed the sensory stimuli and environments that autistic individuals liked or disliked, the impact of these stimuli on physical and mental conditions, and coping strategies (10, 11, 28, 29). Some self-reports from individuals with ASD or their online communities revealed their detailed “feelings” in response to sensory inputs. They considered their perception quite different from that of typically developed individuals and doubted whether they were seeing the world in the same way (29). Concrete patterns of atypical perception, such as seeing everything in high contrast or hearing white noise that does not exist in the environment, have been reported. These reports also indicated that specific patterns of atypical perception occur in particular scenarios. For example, some individuals with ASD have reported that their visual or auditory noise perceptions often occur in crowded places. Unlike color blindness and other permanent sensory impairments, this kind of atypical perception seemed to diminish when the environment changed to a peaceful or familiar place. Therefore, we hypothesized that audio-visual stimuli in the environment are critical factors in atypical perception induction.

There is a lack of studies that systematically investigate the detailed patterns of atypical perception in ASD. This is partly due to the difficulty of describing the inner sensory experience literally. Another challenge is evaluating the environmental causes of atypical perceptions. Quantitative approaches in behavioral studies focused on participants’ responses to sensory stimuli and were insufficient to access the inner experience of perception. Data collected using a qualitative approach are inappropriate for the statistical analysis of the relationship between atypical perception and environmental factors. In this study, we applied a systematic approach to assist participants in reporting their sensory experiences quantitatively, aiming to reveal detailed patterns of atypical perception and their association with audio-visual stimuli. We expect these results to help us infer the underlying mechanisms of sensory processing in ASD.

## Materials and Methods

Our research group has proposed an approach that quantitatively analyzes self-reports from individuals with ASD and applied it to investigate their atypical visual perception (30). This study adopted a similar approach to investigate auditory perception. Signal processing methods (SPMs) were prepared to simulate potential patterns of atypical auditory perception for individuals with ASD. Participants were asked to select the SPMs and adjust their strength to make the audios similar to their daily experiences. The strength of the SPMs was quantitatively recorded, which was feasible for further statistical analyses. Finally, we applied principal component regression analysis to evaluate the relationship between the audio-visual features of the input videos and the strength of the selected SPMs.

### Participants

Twenty-five high-functioning autistic adults with auditory hyperesthesia/hypoesthesia (14 men and 11 women) and 25 typically developed adults (TD; 15 men and 10 women) were recruited from Tokyo and Osaka, Japan. The age of the ASD group ranged from 18 to 52 years (mean = 32, *SD* = 10), and that of the TD group ranged from 21 to 57 years (mean = 34, *SD* = 18). All the participants in the ASD group had been diagnosed with ASD or Asperger’s syndrome (*n* = 12) at a medical institution. They also reported having experienced auditory hyperesthesia/hypoesthesia. We used the Autism Spectrum Quotient (31) to assess their autistic traits [mean score (SD): 38 (7), range: 22–48], which was significantly higher [*t*(42) = 10.9, *p* < 0.001] than that of the TD group [mean score (SD): 17.9(6), range: 9–31]. The participants were required to be computer-literate and able to recall their auditory experiences. We excluded three participants each in the ASD and TD groups due to low data reliability (see the “Data Analysis” section for further details). Another group of 11 adults with high-functioning ASD and auditory hyperesthesia/hypoesthesia participated in our preliminary experiment. Their ages ranged from 30 to 59 years (mean = 43.1). All participants provided written informed consent. The study was reviewed and approved by the Institutional Review Boards of Osaka University (Osaka, Japan), the University of Tokyo (Tokyo, Japan), and the National Institute of Information and Communications Technology (Tokyo, Japan).

### Preliminary Experiment

We conducted a preliminary experiment to determine the patterns of atypical auditory perception to be examined in the main experiment. We used audio signal processing techniques to prepare 22 prototypes of SPMs to imitate the potential patterns of auditory perception based on previous self-reports from individuals with ASD. The SPMs included those that manipulate the amplitude of sounds (amplifier, attenuator, attenuator on one ear, high-pass filter, low-pass filter, band-pass filter, band-reject filter, fade-out effect, and fade-in effect), adjust the tempo or frequency of sounds (delay on one ear, speeding up the tempo, slowing down the tempo, increasing and decreasing fundamental frequencies), add additional sounds (adding noise and adding a single tone), and create special sound effects (flanger, phaser, water effect, telephone effect, echo effect, and repeating sounds). The experimenters first showed videos of a few scenarios (busy street, TV news, train station, beach, café, and fireworks), without any SPM. The participants then watched the videos influenced by each SPM. We asked the participants to judge whether the effects of the SPMs were similar to their auditory experiences in these scenarios. The SPMs judged as experienced by more than half of the participants were used in the main experiment.

### Signal Processing Methods as Mimickers of Potential Atypical Perception

Six SPMs were determined from the preliminary experiment, and their strengths were adjustable throughout the experiment. The details were:

(1) An SPM-amplifier to increase the intensity of the original sounds. The strength ranged from no enhancement to the full output volume of the Mac audio driver.
(2) An SPM-noise to add white noise. The strength was determined by the output volume of the white noise, which ranged from zero to one-half of the full volume.
(3) An SPM-single-tone to add a sine wave tone. Two parameters were adjustable: The output volume of the tone, which ranged from 0 to 7% of the full volume, and the frequency of the tone, which ranged from 500 to 5,000 Hz.
(4) An SPM-band-reject to attenuate sounds within a particular frequency range. Two parameters were adjustable: the width of the rejected band, which ranged from 0 to 10,000 Hz, and the center frequency of the band, which ranged from 5,000 to 10,000 Hz.
(5) An SPM-echo to copy the original sounds with a delay and decay in intensity and then overlap the original sounds with the copy. The decay rate was 40% per second. The delay of the copied sounds was adjustable in the range of 0–1,500 ms.
(6) An SPM-flanger to copy the original sounds with a small and gradually changing delay, and then mix the original sounds with the copy. The period of one cycle of delay was adjustable over the range 0.1–10 s.

The original sounds were derived from videos that presented various daily scenarios. We recorded videos in 30 situations (e.g., busy street, train platform with a train passing, peaceful park) that covered a wide range of audio-visual intensities. Supplementary Table 1 and Supplementary Figure 1 provide detailed information about the audio-visual intensity of the video clips. Each video clip was 20 s long.

### Procedure

**FIGURE 1:**
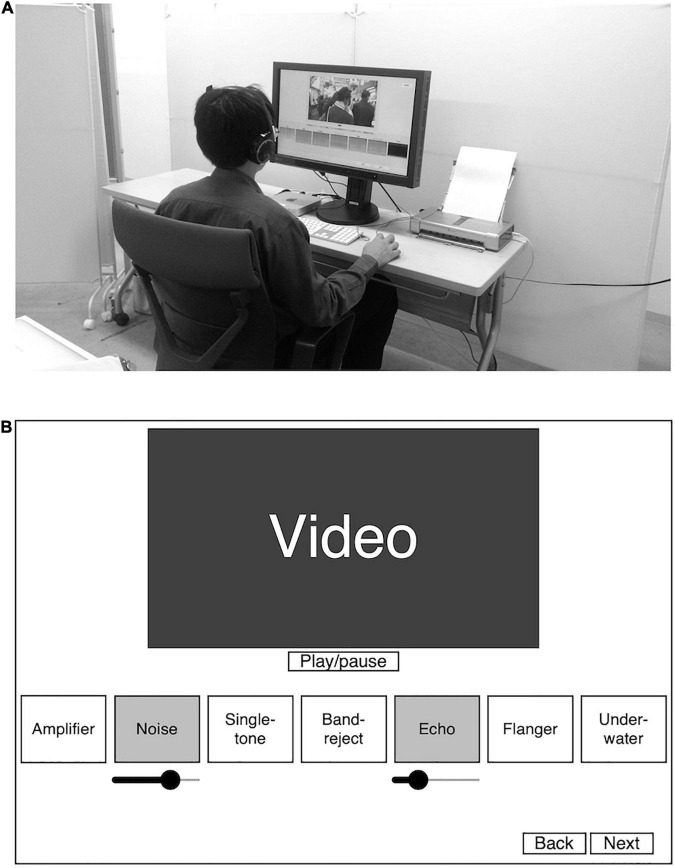
The environmental setting of the experiment **(A)** and the interface used in the experiment **(B)**.

Participants heard the sound modified by the SPMs, compared it to the sound in their memory, and adjusted the strengths of SPMs to make the modified sound close to their auditory experiences as much as possible. Figure 1A shows the environmental settings of the experiment. The participants used a Mac OS desktop computer to watch the videos and respond. A Sennheiser U320 headphone and a 27-inch liquid-crystal display were used to present the audio signals and video clips. We arranged a white partition or a white wall behind the display in order to reduce distraction. Each participant adhered to the following procedures.

(1) Watching a video:

A video clip was shown in full-screen mode. The participant heard the original sound that was not affected by any SPM. The participant was instructed to recall the auditory experience in the scenario shown by the video.

(2) A. Checking SPMs’ effects:

Once the video clip ended, the full-screen mode would be closed, and an interface such as that shown in Figure 1B was presented. The video clip was played in a loop in the top panel of the interface, while buttons of the SPMs were presented below the video screen. In this step, the controllers under the buttons of SPMs were not shown. SPMs were applied to the video clip as soon as the participant clicked the corresponding buttons. Thus, the participant could immediately check the effects of the SPMs. The selected SPM could be canceled by clicking it again. If more than one SPM were selected, the effects of multiple SPMs would be applied simultaneously.

B. Selecting SPMs:

The participant selected all SPMs that made the sound close to the atypical perception that has been experienced in the given scenario before. If the participant had not experienced atypical perception similar to the effect of any SPM, no SPM was selected. The participant clicked the “Next” button to determine the selection and go to the next step.

(3) Adjusting individual SPM’s strength:

Only one controller of the selected SPMs was shown on the interface at a time. The participant used the controller to adjust the SPM’s strength until the sound became similar to their experience. After the adjustment was finished, the participant clicked the “Next” button. This step was repeated for each selected SPM. If no SPM was selected, this step would be skipped.

(4) Adjusting multiple SPMs’ strengths:

The participant checked the combined effect if they selected more than one SPM. The controllers of all selected SPMs were presented on the interface, and the participants could adjust them simultaneously. The participant finished this step by clicking the “Next” button.

(5) Ending one trial:

The interface returned to step (1) with the next video clip.

During steps from (2) to (4), the participant could click the “Back” button to return to the previous step.

Two video clips, which contained intense audio-visual stimuli and might trigger atypical perception, were presented twice to check the reliability of the participants’ answers; the “Data Analysis” section presented below describes the details of the reliability calculation. The other videos were presented only once to each participant. The video clips were presented in a pseudo-random order, except for the first trial, which used the clip taken on a bus. We chose it as the first trial because the effects of all the SPMs were obvious on this clip. The first repeated video clip was used once in the 2nd to 8th trials and once in the 17th to 24th trials. The second repeated video was used once in the 9th to 16th trials and once in the 25th to 32nd trials. In the end, we obtained a record of responses to the 6 SPMs for each of the 32 trials.

The time required to complete the experiment varied among participants. The approximate time range was 40 min to 2 h. Participants were allowed to rest at any time during the experiment. The experimenter also suggested that the participants pause the experiment depending on their condition.

### Data Analysis

We extracted three visual and five auditory features from the videos to analyze their relationship with atypical perception. The visual features included:

(1) Brightness;
(2) Movement (i.e., the change in brightness between consecutive image frames);
(3) Complexity (i.e., the number of edges detected in the image).

The auditory features included:

(1) The sound level (dB SPL) of the whole sound (i.e., the full range of frequencies);
(2) The sound level of low-frequency sounds (20-200 Hz);
(3) The sound level of mid-frequency sounds (200-2,000 Hz);
(4) The sound level of high-frequency sounds (2,000-20,000 Hz);
(5) The center frequency (i.e., the spectral center of gravity calculated on a linear-frequency scale. It represents the average frequency of the sound spectrum).

We used the speech analysis software Praat (version 6.0.17) (32) to analyze the auditory features. The average values, standard deviations, and changing rates throughout the frames of all eight features were used in the following analysis.

Some audio-visual features were highly correlated (e.g., the sound level of low-, mid-, and high-frequency sounds). Multicollinearity in the multiple regression analysis had a substantial impact. Therefore, this study used principal component regression (PCR) to estimate the relationship between atypical auditory perception and audio-visual features. The first step was to perform principal component analysis (PCA) to obtain the principal components of the audio-visual features. Only the principal components that explained 95% of the features were used for further calculations. We then adopted simple linear regression and used the principal components as explanatory variables and strengths of the SPMs as dependent variables. The final step was to transform regression coefficients to the scale of the audio-visual features based on PCA loading and then obtain a regression model of auditory perception on audio-visual features. The multicollinearity problem was addressed by excluding low-variance principal components. All statistical analyses were conducted using R (version 3.6.3) (R Foundation, Vienna, Austria).

We compared the results of the repeated video clips to check the reliability of the participants’ answers. If participants reported their perceptions accurately, their responses to the repeated videos would have been consistent. Two criteria were used to judge reliability: first, the ratio of inconsistently selected SPMs, which were selected only once in the two trials of the same repeated video clip, to all selected SPMs. The second criterion was the correlation between the strengths adjusted in the trials with the same repeated video clip. We excluded the data of participants who had a ratio value larger than 0.5 and a correlation value lower than 0.5.

### Sensory Profile

We used the Adolescent/Adult Sensory Profile (AASP) (33, 34) to compare the sensory characteristics of the ASD and TD groups. The AASP is intended to evaluate behavioral responses to daily sensory experiences. It has four quadrants: Low registration, sensation seeking, sensory sensitivity, and sensation avoiding, based on the intersections of a sensory threshold factor (i.e., high or low) and behavioral response factor (i.e., active or passive). Low registration includes diminished responses to sensations, such as missing stimuli. Sensation seeking represents active behavior to pursue more intense sensory stimuli. Sensory sensitivity reflects responses like distractibility and discomfort with sensory stimuli. Sensation avoiding includes behaviors that reduce exposure to stimuli, such as preventing exposure to unfamiliar or intense stimuli.

## Results

All the results presented in this section were obtained from the 44 participants recruited in the main experiment.

### Sensory Profiles

**FIGURE 2:**
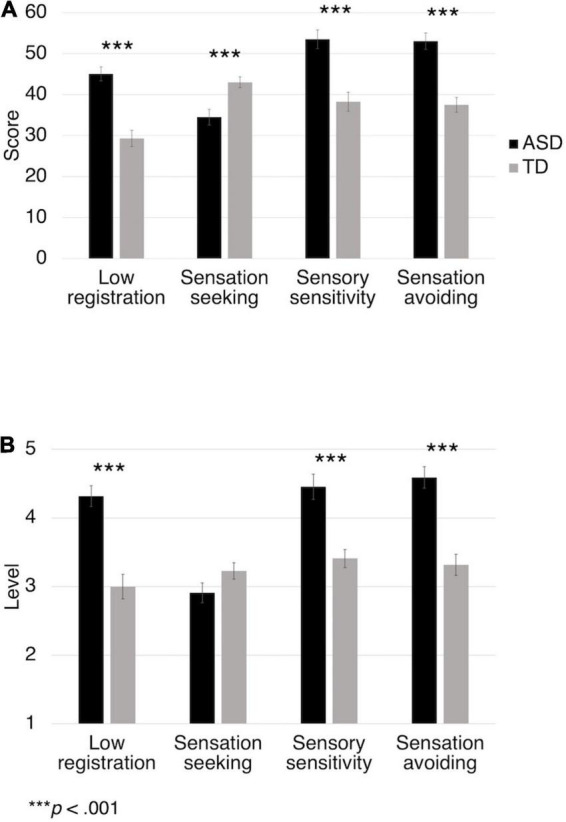
The results of the Adolescent/Adult Sensory Profile. The raw scores are shown in **(A)**. The scores are classified into five levels for comparison with most people in **(B)**. The levels from 1 to 5 represent much less than most people, less than most people, similar to most people, more than most people, and much more than most people, respectively.

The AASP results confirmed that the ASD group had more severe sensory issues. The scores of the ASD and TD groups were significantly different for all four quadrants (Figure 2A). The ASD group scored higher on low registration [*t*(42) = 6.7, *p* < 0.001], sensory sensitivity (*t* = 5.1, *p* < 0.001), and sensation avoiding (*t* = 6.2, *p* < 0.001) and lower on sensation seeking (*t* = 3.9, *p* < 0.001). The AASP manual provides a system for classifying raw scores into five levels, from much more to much less than most people. Figure 2B shows that the TD group had sensory behavior levels similar to those of most people. Additionally, the ASD group had more behaviors of low registration (*t* = 6.2, *p* < 0.001), sensory sensitivity (*t* = 4.7, *p* < 0.001), and sensation avoiding (*t* = 6.4, *p* < 0.001) than the TD group, but the difference in sensation seeking was not significant (*t* = 1.8, *p* = 0.072).

### Atypical Auditory Perception

**FIGURE 3:**
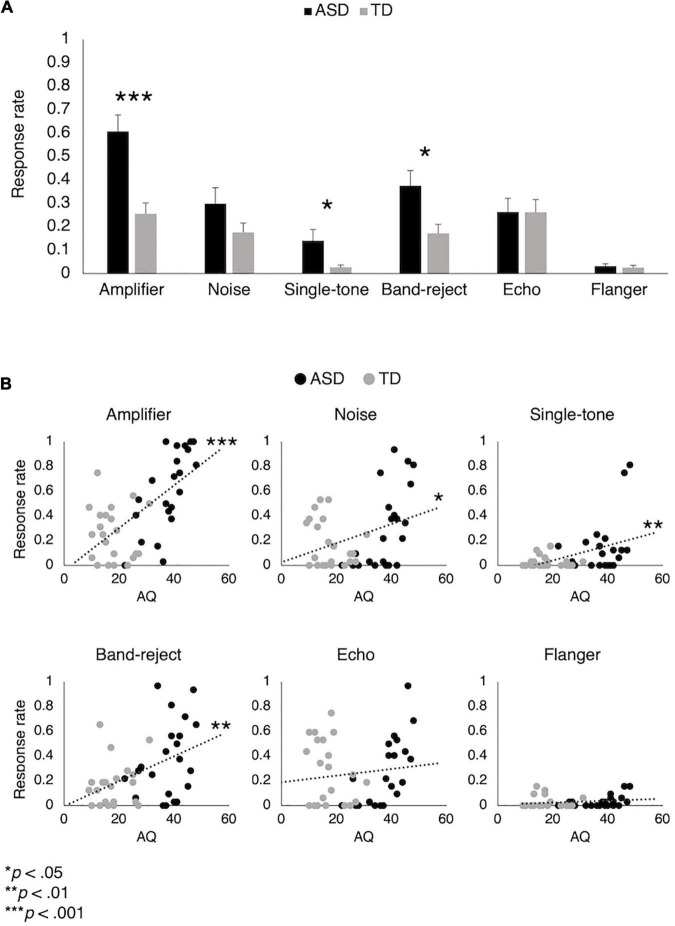
The response rates for the six patterns of atypical auditory perception, which represent how often each signal processing method was selected by the participants. Comparison of the ASD and TD groups is shown in **(A)**. The ASD group selected the SPM of amplifier, single-tone, and band-reject more frequently than the TD group. The distribution of response rates along with autistic traits are shown in **(B)**. There is a trend that response rates are positively correlated with autistic traits.

Figure 3 shows the response rates for the SPMs. The response rate was calculated for each of the SPMs and defined as the number of trials in which a participant selected that SPM divided by the total of 32 trials. Atypical auditory perception was found for both the ASD and TD groups. However, the ASD group generally had higher response rates. We used the Mann-Whitney *U*-test to assess the differences in response rates between the groups because the data did not fit the assumption of normality. The response rates for the amplifier (*U* = 390.0, *p* < 0.001), band-reject (*U* = 345.0, *p* = 0.016), and single-tone (*U* = 326.5, *p* = 0.035) were significantly higher for the ASD group. The response rate to noise was also higher for the ASD group, but the difference was not significant (*U* = 276.0, *p* = 0.425). Although the echo occurred quite frequently, the response rates for the groups were comparable (*U* = 236.0, *p* = 0.896). The response rates for the flanger were quite low, indicating the rarity of this pattern among the participants. Therefore, we do not discuss the regression results for the flanger in the following sections. Finally, the response rates exhibited by the TD group were higher than those expected. This may be due to the properties of the task. Even if participants experienced a certain auditory perception only a few times, they were asked to select the corresponding SPM.

Figure 3B shows the relationship between the response rates and autistic traits. There was a trend of positive correlations, especially for the amplifier [*r*(42) = 0.65, *p* < 0.001] and band-reject (*r* = 0.46, *p* = 0.002). The correlation with SPM-noise was significant but relatively weak (*r* = 0.35, *p* = 0.019). The significant correlation with single-tone (*r* = 0.44, *p* = 0.003) was influenced by outliers; it became marginally significant (*r* = 0.30, *p* = 0.053) after excluding outliers. Similar to the results of the Mann-Whitney *U*-test, SPM-echo was not correlated with autistic traits (*r* = 0.12, *p* = 0.421).

A concern is that the correlations may have been affected by group differences in addition to autistic traits. Unlike the TD group, all the participants in the ASD group had experienced hyperesthesia/hypoesthesia, which may have been associated with the atypical auditory perception found in the experiment. To clarify this concern, we conducted a hierarchical regression analysis on the four types of SPMs that were correlated with AQ (Autism Spectrum Quotient). For each type, we built three regression models by adding an independent variable at each step. AQ was the only independent variable in the first regression model. Group was added to the second model, and the interaction between AQ and group was the added variable in the last step. We checked whether the added variables improved the proportion of variance in the response rates explained by the model (*R*^2^). The *R*^2^ values were not significantly improved by group for all four types of SPM (amplifier: Δ*R*^2^ < 0.01, *p* = 0.652; band-reject: Δ*R*^2^ < 0.01, *p* = 0.899; noise: Δ*R*^2^ < 0.01, *p* = 0.836; single-tone: Δ*R*^2^ < 0.01, *p* = 0.578), which indicates that group differences do not explain more variation than AQ. The interaction between AQ and group significantly improved *R*^2^ for the response rates to amplifier and noise (amplifier: Δ*R*^2^ = 0.10, *p* = 0.012, noise: Δ*R*^2^ = 0.13, *p* = 0.021) but not those for band-reject and single-tone (band-reject: Δ*R*^2^ = 0.01, *p* = 0.587, single-tone: Δ*R*^2^ = 0.02, *p* = 0.374). Moreover, AQ did not significantly contribute to the last regression model. These results confirm that autistic traits increase the occurrence of atypical auditory perception; however, this trend was mainly found in the ASD group. Supplementary Table 2 summarizes the results of the hierarchical regression.

### Correlation of Audio-Visual Features With Atypical Perception

We adopted the 1st to 10th principal components in the PCR analysis. The results for each auditory SPM are summarized below. Supplementary Tables 3, 4 show the details of all the regression results.

#### Correlation of Amplifier Perception With Sound Level

**FIGURE 4:**
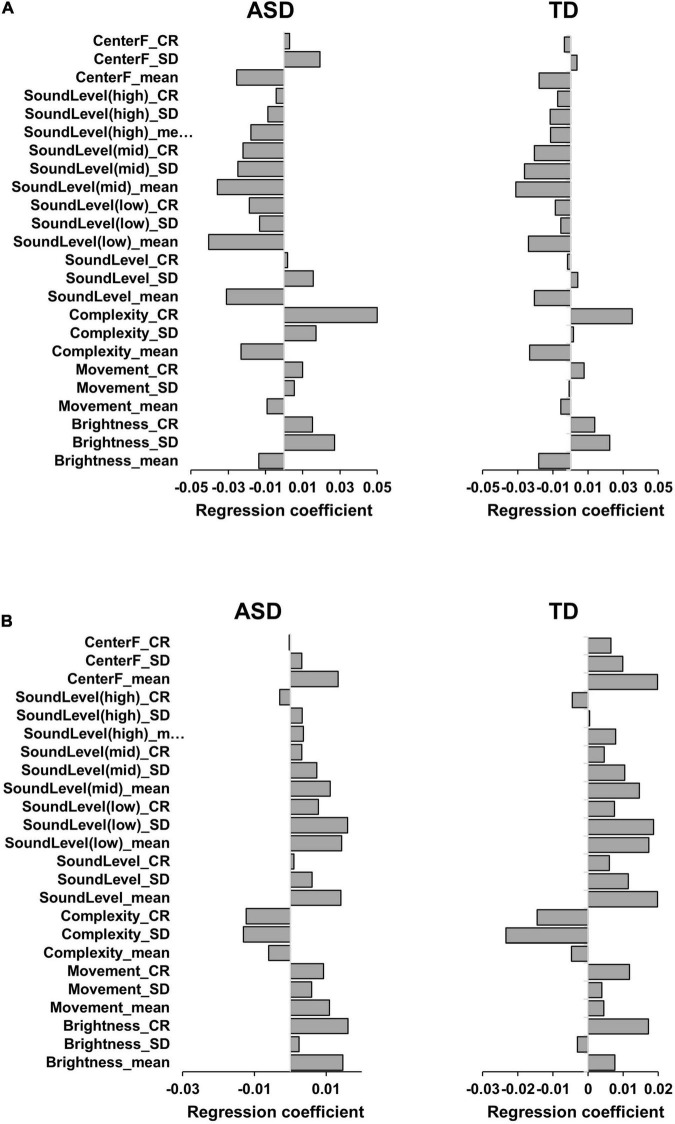
The regression coefficients of the principal component regression models for the amplifier **(A)** and noise perception **(B)**. SD, CR, and centerF mean standard deviation, changing rate, and sound center frequency, respectively.

Regression analysis revealed significant interactions between the amplifier and principal components of the audio-visual features in both the ASD (adjusted *R*^2^ = 0.023, *F* = 2.64, *p* = 0.004) and TD (adjusted *R*^2^ = 0.048, *F* = 4.52, *p* < 0.001) groups. Figure 4A shows the regression coefficients that characterize the regression models. The patterns of the models were similar for the two groups. Furthermore, the levels of the sounds, including those with low- and mid-frequencies, had stronger negative regression coefficients. Other features, such as the mean value and changing rate of complexity, also contributed to the amplifier. These results imply that amplifier perception occurs in quiet environments.

#### Correlation of Noise Perception With the Level and Fluctuation of Stimuli

The regression models for SPM-noise fitted the data significantly for both the ASD (adjusted *R*^2^ = 0.014, *F* = 2.02, *p* = 0.029) and TD (adjusted *R*^2^ = 0.039, *F* = 3.88, *p* < 0.001) groups. Similar to the amplifier results, the regression models for the two groups were similar to a certain extent (Figure 4B). The results for the ASD group indicated that SPM-noise was correlated with the brightness, sound level, and changes in the audio-visual stimuli, such as the mean value and changing rate of brightness, the mean value of the sound level of the whole-frequency sounds, and the mean value and standard deviation of the sound level of low-frequency sounds. The TD group had stronger regression coefficients for the mean value and standard deviation of the sound level but not the mean brightness. The models for both groups had negative coefficients for the features of complexity.

#### Correlation of Echo With Movement and Variation in Sound Level

The regression model for the ASD group significantly explained the variance in the strength of echo (adjusted *R*^2^ = 0.020, *F* = 2.39, *p* = 0.009). The regression coefficients of features such as the mean value and standard deviation of mid-frequency sound level, the standard deviation of high-frequency sound level, and the mean value and standard deviation of movement, were larger (Supplementary Table 3). In contrast, we did not find a significant model for the SPM-echo data of the TD group (adjusted *R*^2^ = 0.001, *F* = 1.10, *p* = 0.363).

#### Band-Reject

Regression analysis did not reveal a relationship between band-reject and audio-visual features. The regression models for the width (ASD: adjusted *R*^2^ = 0.008, *F* = 1.58, *p* = 0.110; TD: adjusted *R*^2^ = –0.001, *F* = 0.95, *p* = 0.489) and frequency (ASD: adjusted *R*^2^ = 0.008, *F* = 1.60, *p* = 0.103; TD: adjusted *R*^2^ = –0.004, *F* = 0.75, *p* = 0.682) of the rejected bands were not significant.

#### Single-Tone

The regression model was not significant for the volume (adjusted *R*^2^ = 0.004, *F* = 1.32, *p* = 0.218) or frequency (adjusted *R*^2^ = –0.01, *F* = 0.46, *p* = 0.913) of SPM-single-tone for the ASD group. The models of the single-tone perception of the TD group, which had a very low response rate, were also insignificant (volume: adjusted *R*^2^ = 0.001, *F* = 1.04, *p* = 0.471; frequency: adjusted *R*^2^ = –0.001, *F* = 0.94, *p* = 0.497).

It should be noted that the *R*^2^-values were small in all regression models. Other factors, like individual differences, may also be important for determining the strength of SPMs.

### Correlation of Atypical Perception With Adolescent/Adult Sensory Profile

We performed a correlation analysis on response rates of SPMs with the results of AASP to check how atypical perception interrelates with the categories of sensory profiles. Supplementary Table 5 summarizes the results. After using the Bonferroni correction, we found significant correlations for SPMs of amplifier, single-tone, and band-reject. The response rate of SPM-amplifier was significantly correlated with low registration [*r*(42) = 0.46, *p* = 0.0016], sensory sensitivity [*r* = 0.68, *p* < 0.0001], and sensation avoiding (*r* = 0.62, *p* < 0.0001). There was a positive correlation of SPM-single-tone with sensation avoiding (*r* = 0.45, *p* = 0.0023). The correlation of SPM-band-reject with low registration was marginally significant (*r* = 0.44, *p* = 0.0026). The results implied that, separately, single-tone perception and band-reject perception can be considered as symptoms of sensation avoiding and low registration. In addition, amplifier perception may occur in people with different types of sensory profiles. However, more evidence is required to clarify the relationship between the atypical perceptions and sensory profile.

## Discussion

This study showed that the occurrence of the amplifier, single-tone, noise, and band-reject perceptions were positively correlated with autistic traits, especially for the ASD group. We demonstrated that the audio-visual features related to the SPMs simulating atypical perception and found that the amplifier and noise perception of the ASD and TD groups occurred in similar environments, implying that the same causes were shared by the two groups. The mechanism of atypical perception may be similar in individuals with and without autism, but it is easier to trigger in individuals with higher autistic traits. Future studies are required to investigate the factors that increase the occurrence of atypical perceptions. The following sections compare our results with the findings of previous studies on atypical perception, including that of the general population, to discuss the potential underlying mechanisms.

### Potential Mechanisms of Atypical Perceptions

#### Noise and Single-Tone: Tinnitus

Noise and single-tone perceptions can be considered types of tinnitus, that is, hearing a sound that does not exist in the environment. Tinnitus is more prevalent in individuals with Asperger’s syndrome (35). Even in the general population, more than 10% had experienced tinnitus lasting for more than 5 min in the past year (36, 37). Hence, it was not surprising that the TD participants had experienced noise or single-tone perception.

The development of tinnitus is multifactorial (38). The most common causes are hearing loss and noise exposure (39, 40). Cumulative exposure to excessive noise is recognized as a cause of permanent tinnitus. Additionally, temporary noise exposure can trigger temporary tinnitus (41, 42). Emotional stress is also associated with tinnitus (43, 44). Nevertheless, the trigger for tinnitus sometimes cannot be identified (41, 45). Our finding of a correlation between SPM-noise and sound level agrees with reports that exposure to intense sound can induce tinnitus. Other audio-visual features related to SPM-noise, such as the variance of sound level, may be possible triggers of tinnitus in both autistic and general populations.

#### Band-Reject: Deficit of Cochlear Function

The higher hearing threshold at mid-range frequencies (2 kHz) in individuals with ASD is associated with their speech abilities (46). Another study on otoacoustic emission found reduced outer hair cell function around the 1-kHz mid-frequency region in children and adolescents with ASD (47). Outer hair cells help humans detect a sound in the presence of another sound with a different frequency (48, 49), which is critical to the sophisticated skills of speech or music perception. The central frequency of the band-reject of the ASD group in our experiment was approximately 2 kHz. This is consistent with mid-frequency hearing difficulties reported in previous studies (46, 47). Band-reject perception may be explained by impairment of the outer hair cells or associated cochlear function.

## Conclusion

This study investigated atypical auditory perception and associated it with audio-visual features by quantitatively evaluating self-reports of sensory experiences. Participants with ASD selected SPMs of amplifier, band-reject, and single-tone perceptions more often than controls. The results of the regression analysis indicate that amplifier perception occurs in an environment with a low sound level, noise perception is induced by intense and unsteady audio-visual stimuli, and echo perception is correlated with movement and variation in sound level. The environmental causes of amplifier perception and the causes of noise perception were similar for the ASD and TD groups. The results help us infer the potential mechanism underlying atypical perceptions. Overall, we suggest that sensory processing in ASD follows a mechanism similar to that in TD; however, some over- or under-neural functioning enhances the probability of triggering atypical perception. In addition, the findings of environmental causes are useful for creating an autism-friendly environment. Individuals with ASD could also refer to these results to reduce the influence of atypical auditory perception by avoiding the causes.

### Limitations of This Study

This study focused on atypical perceptions that vary according to the environment. Auditory difficulties with a continuous influence, such as permanent hearing loss, were not the targets of this study. If permanent hearing difficulty interacts with variable one, the participant’s experience may deviate significantly from the auditory effect produced by our SPMs. We are unable to deny this possibility, but the similar regression results for the ASD and TD groups imply that the two groups had the same types of atypical perception. Since TD participants are not supposed to have permanent hearing difficulty, we suggest that the atypical perception found in the ASD group was also not affected by permanent hearing difficulty.

The recruitment of participants to the ASD group was restricted to those who had auditory hyperesthesia/hypoesthesia. It should be noted that not every individual with autism has the atypical auditory perceptions found in this study. Hyperesthesia/hypoesthesia may be strongly associated with atypical auditory perception. For instance, the prevalence of hyperacusis in individuals with ASD was approximately 27% in a meta-analysis (50); however, 13 of the 22 participants in our ASD group had response rates to hyperacusis (amplifier) larger than 0.5. The proportion of individuals with atypical auditory perception in the entire ASD population may not be as large as that in our participants.

SPMs, such as amplifiers that produce intense sounds, may disturb participants and affect their perception. A few participants reported disliking the SPM-amplifier effect. We carefully monitored the state of the participants during the experiment and provided them with enough rest to ensure that the burden caused by the SPMs was minimal and that they were in a comfortable condition.

Another concern regarding our experimental design was the demand characteristics. The task was to reproduce atypical perceptions; therefore, the participants may tend to report more than they experienced. This may be one of the reasons why the response rate in the TD group was not very small. To reduce the effect of demand characteristics, we told the participants not to respond if they did not experience any atypical perceptions. Furthermore, the effect of demand characteristics alone is insufficient to explain the difference in response rates between groups and between SPMs.

It needs to be noted that the diagnostic criteria for participants in the ASD group might be inconsistent. Although the participants claimed that they were diagnosed with ASD or Asperger’s syndrome at a medical institution, the medical institutions might use different diagnostic criteria. In addition, we did not know the criteria as well as their reliabilities. However, we used the Autism Spectrum Quotient to assess their autistic traits and confirmed that their AQ is significantly higher than that of the TD group.

## Data Availability Statement

The raw data supporting the conclusions of this article will be made available by the authors, without undue reservation.

## Ethics Statement

The studies involving human participants were reviewed and approved by the Institutional Review Boards of Osaka University, The University of Tokyo, and the National Institute of Information and Communications Technology. The patients/participants provided their written informed consent to participate in this study. Written informed consent was obtained from the individual(s) for the publication of any potentially identifiable images or data included in this article.

## Author Contributions

YN, S-IK, SA, and MA contributed to the study’s conception and design. J-JH, YN, and S-IK conducted the experiments. J-JH performed the statistical analyses and drafted the manuscript. J-JH, YN, and MA interpreted the results. All authors contributed to manuscript revision, read, and approved the submitted version.

## Conflict of Interest

The authors declare that the research was conducted in the absence of any commercial or financial relationships that could be construed as a potential conflict of interest.

## Publisher’s Note

All claims expressed in this article are solely those of the authors and do not necessarily represent those of their affiliated organizations, or those of the publisher, the editors and the reviewers. Any product that may be evaluated in this article, or claim that may be made by its manufacturer, is not guaranteed or endorsed by the publisher.
