# Supplementary material for: Atypical Auditory Perception Caused by Environmental Stimuli in Autism Spectrum Disorder: A Systematic Approach to the Evaluation of Self-Reports
Source: Front Psychiatry. 2022 Jun 9;13:888627. doi: 10.3389/fpsyt.2022.888627 (PMC9236639; doi:10.3389/fpsyt.2022.888627)
Supplement: Supplementary file 1 [file Data_Sheet_1.docx]

Supplementary Material

# Supplementary Tables

| **Supplementary Table 1.** Audio-visual intensities of the video clips | | | | |
| --- | --- | --- | --- | --- |
| **Scenario** | **Brightness (grayscale)** | **Brightness STD** | **Loudness (dB)** | **Loudness STD** |
| Cafeteria | 104.3 | 1.6 | 75.1 | 0.7 |
| City street | 108.1 | 2.5 | 76.5 | 2.1 |
| Coast | 127.1 | 3.5 | 71.8 | 2.7 |
| Crosswalk | 123.9 | 2.5 | 67.7 | 1.0 |
| Crowded street | 106.6 | 11.6 | 75.6 | 1.3 |
| Elevator | 92.4 | 6.1 | 60.6 | 4.9 |
| Firework | 5.2 | 2.2 | 68.3 | 2.6 |
| Forest | 93.9 | 1.0 | 50.2 | 1.9 |
| Forest river | 81.2 | 0.8 | 60.3 | 0.2 |
| Harbor | 131.6 | 1.8 | 60.7 | 1.3 |
| Highway in night | 38.4 | 5.6 | 78.0 | 0.4 |
| In a bus | 86.1 | 6.8 | 71.3 | 2.0 |
| In a train | 127.2 | 11.1 | 69.2 | 1.7 |
| Lake | 127.9 | 0.8 | 50.2 | 2.6 |
| Market | 100.2 | 7.7 | 76.4 | 1.1 |
| Night city | 65.8 | 10.3 | 63.8 | 2.4 |
| Night store | 43.1 | 10.4 | 33.4 | 2.6 |
| Night street | 33.2 | 6.8 | 36.3 | 1.9 |
| Park | 114.9 | 1.1 | 47.6 | 1.4 |
| Peaceful street | 109.3 | 1.9 | 48.2 | 4.0 |
| Raining | 129.4 | 0.8 | 65.0 | 1.7 |
| Restaurant | 92.4 | 1.9 | 71.0 | 2.6 |
| Road crossing | 119.1 | 4.7 | 64.2 | 2.4 |
| Ski resort | 175.9 | 5.8 | 56.4 | 1.9 |
| Snowing | 81.9 | 10.4 | 74.0 | 1.7 |
| Stair | 114.0 | 14.4 | 64.4 | 2.1 |
| Supermarket 1 | 110.0 | 2.8 | 69.6 | 1.5 |
| Supermarket 2 | 116.8 | 5.1 | 69.4 | 1.9 |
| Train station 1 | 99.7 | 8.3 | 68.3 | 4.0 |
| Train station 2 | 85.6 | 15.1 | 80.1 | 1.1 |

| **Supplementary Table 2.** Results of hierarchical regression analysis | | | |
| --- | --- | --- | --- |
|  | | | |
| (a) Amplifier | | | |
| Predictor variables | Regression 1 | Regression 2 | Regression 3 |
| AQ | 0.49*** | 0.42* | -0.11 |
| Group |  | 1.99 | -24.03* |
| AQ × Group |  |  | 0.98* |
|  |  |  |  |
| R^2^ | 0.34 | 0.34 | 0.45 |
| ΔR^2^ | 0.34 | 0.00 | 0.10* |

| (b) Band-reject | | | |
| --- | --- | --- | --- |
| Predictor variables | Regression 1 | Regression 2 | Regression 3 |
| AQ | 0.31** | 0.29 | 0.17 |
| Group |  | 0.57 | -4.97 |
| AQ × Group |  |  | 0.21 |
|  |  |  |  |
| R^2^ | 0.18 | 0.18 | 0.19 |
| ΔR^2^ | 0.18 | 0.00 | 0.01 |

| (c) Noise | | | |
| --- | --- | --- | --- |
| Predictor variables | Regression 1 | Regression 2 | Regression 3 |
| AQ | 0.13 | 0.16 | -0.30 |
| Group |  | -0.85 | -22.96 |
| AQ × Group |  |  | 0.84* |
|  |  |  |  |
| R^2^ | 0.04 | 0.04 | 0.17 |
| ΔR^2^ change | 0.04 | 0.00 | 0.13* |

| (d) Single-tone | | | |
| --- | --- | --- | --- |
| Predictor variables | Regression 1 | Regression 2 | Regression 3 |
| AQ | 0.07* | 0.05 | -0.00 |
| Group |  | 0.68 | -1.77 |
| AQ × Group |  |  | 0.09 |
|  |  |  |  |
| R^2^ | 0.14 | 0.14 | 0.16 |
| ΔR^2^ change | 0.14 | 0.00 | 0.02 |

Note. * *p* < .05; ** *p* < .01; *** *p* < .001

| **Supplementary Table 3.** Coefficients table of the principal component regression model (ASD) | | | | | | | | |
| --- | --- | --- | --- | --- | --- | --- | --- | --- |
|  | Amplifier** | Noise* | Echo* | Band-reject  (Width) | Band-reject  (Frequency) | Single-tone  (Volume) | Single-tone  (Frequency) |  |
| Intercept | 0.261 | 0.069 | 0.095 | 0.200 | 0.238 | 0.020 | 0.090 |  |
| Brightness |  |  | | | | | |  |
| Mean | -0.014 | 0.015 ^§^ | 0.006 | 0.044 | 0.054 | 0.001 | 0.007 |  |
| SD | 0.027 | 0.002 | 0.005 | 0.007 | 0.006 | 0.000 | -0.001 |  |
| CR | 0.015 | 0.016 ^§^ | 0.018 | 0.003 | -0.003 | 0.003 | -0.008 |  |
| Movement |  |  |  | | | | |  |
| Mean | -0.009 | 0.011 | 0.021 ^¶^ | 0.017 | 0.014 | 0.001 | -0.002 |  |
| SD | 0.005 | 0.006 | 0.023 ^¶^ | 0.000 | -0.007 | 0.004 | 0.004 |  |
| CR | 0.010 | 0.009 | 0.015 | -0.010 | -0.021 | 0.008 | 0.006 |  |
| Complexity |  |  |  | | | | |  |
| Mean | -0.023 | -0.006 | -0.002 | -0.010 | -0.012 | 0.005 | 0.018 |  |
| SD | 0.017 | -0.013 | 0.010 | 0.019 | 0.022 | -0.006 | 0.007 |  |
| CR | 0.050 | -0.012 | -0.009 | -0.023 | -0.029 | 0.007 | 0.017 |  |
| Sound level |  |  | | | | | |  |
| Mean | -0.031 ^‡^ | 0.014 ^§^ | 0.014 | 0.011 | 0.004 | 0.005 | -0.003 |  |
| SD | 0.016 | 0.006 | 0.005 | 0.011 | 0.017 | -0.002 | -0.006 |  |
| CR | 0.002 | 0.001 | -0.007 | 0.005 | 0.008 | 0.004 | 0.009 |  |
| Sound level  (low-frequency) |  |  | | | | | |  |
| Mean | -0.041 ^‡^ | 0.014 ^§^ | 0.014 | 0.012 | 0.008 | 0.003 | -0.006 |  |
| SD | -0.013 | 0.016 ^§^ | 0.011 | 0.023 | 0.026 | -0.002 | -0.012 |  |
| CR | -0.019 | 0.008 | 0.000 | 0.018 | 0.023 | 0.001 | -0.001 |  |
| Sound level  (mid-frequency) |  |  | | | | | |  |
| Mean | -0.036 ^‡^ | 0.011 | 0.020 ^¶^ | 0.007 | 0.011 | 0.003 | -0.006 |  |
| SD | -0.025 | 0.007 | 0.022 ^¶^ | 0.020 | 0.022 | 0.000 | -0.003 |  |
| CR | -0.022 | 0.003 | 0.009 | 0.019 | 0.021 | 0.004 | 0.008 |  |
| Sound level  (high-frequency) |  |  | | | | | |  |
| Mean | -0.018 | 0.004 | 0.008 | -0.043 | -0.036 | 0.001 | -0.014 |  |
| SD | -0.009 | 0.003 | 0.023 ^¶^ | 0.021 | 0.023 | -0.006 | -0.007 |  |
| CR | -0.004 | -0.003 | 0.009 | 0.012 | 0.014 | 0.000 | 0.007 |  |
| Sound center frequency |  |  | | | | | |  |
| Mean | -0.026 | 0.013 | 0.014 | 0.011 | 0.003 | 0.005 | -0.001 |  |
| SD | 0.019 | 0.003 | 0.006 | 0.005 | 0.006 | 0.000 | -0.002 |  |
| CR | 0.003 | 0.000 | -0.004 | 0.002 | -0.001 | 0.005 | 0.011 |  |
|  | | | | | | | | |

Note. * *p* < .05; ** *p* < .01;

† SD: standard deviation; CR: changing rate.

‡ The regression model of amplifier perception had relatively stronger negative regression coefficients for the sound levels.

§ The correlations of noise perception with the brightness, sound level, and changes in audio-visual stimuli were relatively larger.

¶ Echo perception was correlated with the movement of visual stimuli and variation in the sound level.

| **Supplementary Table 4.** Coefficients table of the principal component regression model (TD) | | | | | | | | |
| --- | --- | --- | --- | --- | --- | --- | --- | --- |
|  | Amplifier*** | Noise*** | Echo | Band-reject  (Width) | Band-reject  (Frequency) | Single-tone  (Volume) | Single-tone  (Frequency) |  |
| Intercept | 0.090 | 0.051 | 0.112 | 0.087 | 0.092 | 0.001 | 0.007 |  |
| Brightness |  |  | | | | | |  |
| Mean | -0.018 | 0.008 | -0.014 | -0.010 | -0.002 | 0.000 | 0.002 |  |
| SD | 0.022 | -0.003 | -0.017 | -0.007 | -0.008 | 0.001 | 0.004 |  |
| CR | 0.014 | 0.017 | 0.000 | -0.007 | -0.006 | 0.001 | 0.002 |  |
| Movement |  |  |  | | | | |  |
| Mean | -0.006 | 0.004 | -0.002 | -0.006 | 0.000 | 0.001 | 0.000 |  |
| SD | -0.001 | 0.004 | 0.001 | 0.001 | 0.003 | 0.001 | -0.003 |  |
| CR | 0.008 | 0.012 | 0.006 | 0.007 | 0.004 | 0.001 | 0.001 |  |
| Complexity |  |  |  | | | | |  |
| Mean | -0.023 | -0.005 | 0.013 | 0.019 | 0.016 | 0.000 | -0.006 |  |
| SD | 0.002 | -0.023 | -0.023 | -0.007 | 0.001 | 0.000 | -0.003 |  |
| CR | 0.035 | -0.015 | -0.025 | 0.010 | -0.004 | 0.002 | 0.006 |  |
| Sound level |  |  | | | | | |  |
| Mean | -0.021^‡^ | 0.020 ^§^ | 0.017 | 0.002 | 0.001 | 0.001 | 0.001 |  |
| SD | 0.004 | 0.011 | 0.000 | -0.007 | -0.011 | 0.000 | -0.002 |  |
| CR | -0.002 | 0.006 | 0.002 | 0.009 | -0.006 | 0.001 | 0.003 |  |
| Sound level  (low-frequency) |  |  | | | | | |  |
| Mean | -0.024 ^‡^ | 0.020 ^§^ | 0.017 | 0.002 | 0.002 | 0.001 | 0.001 |  |
| SD | -0.006 | 0.010 | 0.001 | -0.004 | -0.005 | 0.000 | -0.003 |  |
| CR | -0.009 | 0.007 | 0.006 | 0.010 | 0.000 | 0.001 | 0.001 |  |
| Sound level  (mid-frequency) |  |  | | | | | |  |
| Mean | -0.031 ^‡^ | 0.017 ^§^ | 0.018 | 0.001 | -0.001 | 0.001 | 0.002 |  |
| SD | -0.026 | 0.019 ^§^ | 0.011 | -0.008 | -0.011 | 0.000 | 0.002 |  |
| CR | -0.021 | 0.007 | 0.004 | 0.001 | -0.010 | 0.001 | 0.005 |  |
| Sound level  (high-frequency) |  |  | | | | | |  |
| Mean | -0.012 | 0.015 | 0.002 | -0.005 | -0.010 | 0.000 | 0.002 |  |
| SD | -0.012 | 0.010 | 0.001 | -0.007 | -0.008 | 0.000 | 0.001 |  |
| CR | -0.007 | 0.005 | -0.002 | 0.004 | -0.007 | 0.000 | 0.005 |  |
| Sound center frequency |  |  | | | | | |  |
| Mean | -0.018 | 0.008 | 0.009 | 0.003 | -0.011 | 0.001 | -0.004 |  |
| SD | 0.004 | 0.000 | 0.006 | -0.008 | -0.002 | -0.001 | -0.006 |  |
| CR | -0.003 | -0.005 | -0.001 | 0.004 | -0.004 | 0.000 | 0.000 |  |
|  | | | | | | | | |

Note. *** *p* < .001;

† SD: standard deviation; CR: changing rate.

‡ The regression model of amplifier perception had relatively stronger negative regression coefficients for the sound level.

§ The correlations of noise perception with the sound level were relatively strong.

| **Supplementary Table 5.** Pearson correlation coefficients table of SPMs and AASP | | | | | | |
| --- | --- | --- | --- | --- | --- | --- |
|  | Amplifier | Noise | Single-tone | Band-reject | Echo | Flanger |
| Low registration | 0.46* | 0.32 | 0.32 | 0.44 | -0.01 | 0.12 |
| Sensation seeking | -0.38 | -0.14 | -0.14 | 0.02 | 0.01 | -0.02 |
| Sensory sensitivity | 0.68** | 0.34 | 0.42 | 0.19 | 0.13 | 0.27 |
| Sensation avoiding | 0.62** | 0.29 | 0.45* | 0.36 | 0.20 | 0.18 |

Note. * *p* < .0025; ** *p* < .0001;

# Supplementary Figures


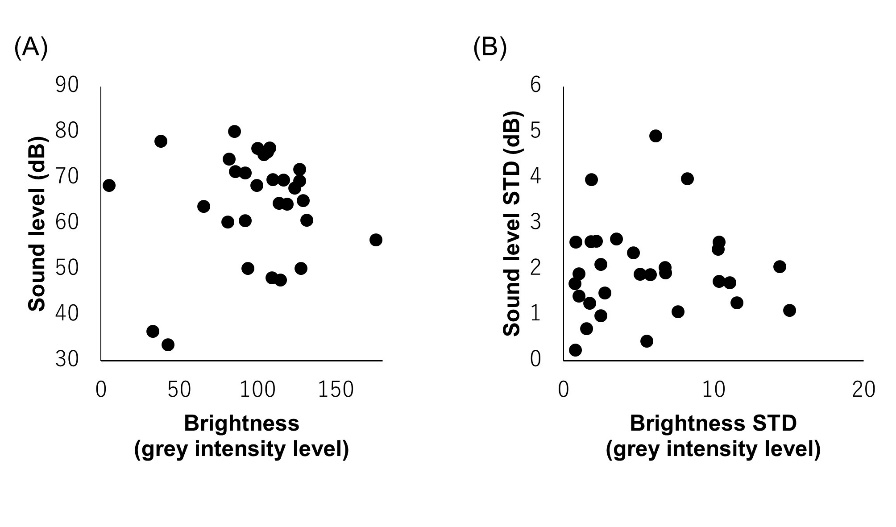


**Supplementary Figure 1.** Distribution of videos along with audio-visual intensities. Brightness was evaluated using grayscale and had a minimum value of 0 and maximum value of 256. The STD represents the standard deviation of all frames of each video clip. The video clips covered a wide range of brightness (5.2 to 175.9) and loudness (33.3 to 80.1 dB).
